# Towards an active inference account of deep meditative deconstruction

**DOI:** 10.1093/nc/niag053

**Published:** 2026-08-24

**Authors:** Shawn Prest, Kevin Berryman

**Affiliations:** Monash Centre for Consciousness and Contemplative Studies, Monash University, 29 Ancora Imparo Way, Clayton, Victoria, 3163, Australia; Monash Centre for Consciousness and Contemplative Studies, Monash University, 29 Ancora Imparo Way, Clayton, Victoria, 3163, Australia

**Keywords:** active inference, cessation, consciousness, deconstruction, meditation, Buddhist, phenomenology, defabrication

## Abstract

This paper explores the intersection of the deep meditative deconstruction resulting from Buddhist meditative practice, specifically through the defabrication process, with the active inference framework (AIF). We propose that defabrication, central to Buddhist meditation, can be understood as a computational process whereby the repeated release of mental tension—associated with clinging and aversion—drives inference progressively lower in an agent’s hierarchical generative model. By casting the release of mental tension as a reduction in hierarchical level-specific precision over beliefs about hidden causes, we demonstrate how the phenomenology of defabrication naturally arises from the resultant system dynamics. This approach enables a computational interpretation of Buddhist meditative states, such as the jhānas, and core concepts like equanimity and stillness. More broadly, it advances a computational phenomenology of meditative deconstruction. Finally, we illustrate the deconstruction process under AIF by extending it to the cessation of phenomenal experience, suggesting this offers insight into core-knowledge structuring and the generation of experience.

## Introduction

A core notion shared by both Buddhism and the active inference framework (AIF) in computational neuroscience (Friston 2010, Parr et al. 2022) is that our experience of reality is in a certain sense constructed. Buddhism holds that all experience is transient, and that all phenomena are fabricated (MN35; Ñāṇamoli 1995). On influential interpretations, AIF implies that phenomenal experience is determined by constructive perceptual inference of the causes of current sensory input; perception can then be cast as ‘controlled hallucination’ (Hohwy and Seth 2020, Clark 2013). This controlled hallucination includes the conscious, phenomenal experience of both self and world, in other words, what it is like to experience ourselves and the world around us. For Buddhists, deep insight into the constructed nature of the self and world distinction is brought to an end with the collapse of the subject/object distinction as part of the process of achieving full awakening, or arahantship, where a lasting collapse of the misrepresentation of perceptual experience (*avijjā*),^1^ results in the end of suffering (Analayo 2008, Harvey 2013). For AIF proponents, the subject/object distinction emerges via Bayesian inference on incoming sense data (Allen and Friston 2018, Apps and Tsakiris 2014). These similarities suggest it may be possible to map Buddhist understandings onto AIF computational mechanisms.

Fabrication (*saṅkhāra*) of phenomenal experience in Buddhism is understood as the construction process by which all sensory input is bound together into an identifiable whole. It arises due to ‘clinging’ to how one is perceiving (Thanissaro, 2020, p. 29; Bodhi, 1980, Walshe,1995: DN 15; Claxon, 1996).^2^ Phenomenologically, clinging produces a palpable feeling of contraction; an active process of subtle mental tightening, constricting, and narrowing in experience, perceived as occurring in a specific area or the entire body (Burbea 2014, Brach 2024, Sucitto n.d.). We refer to this feeling throughout as *mental tension*. Mental tension is experienced as unpleasant and thus has negative *affective valence* (Burbea 2014, Yates et al. 2017).^3^

Experience can systematically be *defabricated* via meditation and insight, understood as a progressive deconstruction process of releasing mental tension, reducing clinging to phenomena in experience, which unbinds previously unified wholes, dis-integrating phenomena into constituent parts (Burbea 2014, Bodhi 2000: SN 46.1). Defabrication results in more positive affective valence as the mental tension is reduced. For example, consider a meditator perceiving a visual object who then notices a subtle pressure behind their eyes. They attempt to relax and let go of this tension. If successful, clinging reduces, and a sense of increased calm arises—the same area now feels lighter, less solidified, less tense, and more spacious. As attention returns to the visual object, it seems less reified. They further notice an increased sense of being in the ‘here and now’ and a more pleasant overall experience.

Canonical cases of defabricated states of experience are meditative absorptions known as the jhānas, which are claimed to be devoid of form or to feature infinite contentless space, and cessations, where perceptual experience utterly ceases. These states are claimed to be accessible to contemporary practitioners of Buddhist meditation. An emerging field of scientific investigation using AIF is beginning to examine these processes (Laukkonen et al. 2023, Chowdhury et al. 2023, Yang et al. 2023) but much speculation remains regarding the phenomenology and cognitive mechanisms of defabrication (Dahl et al. 2015, Brandmeyer et al. 2019, Schoenberg and Vago 2019, Nave et al. 2021, Repetti 2022).

Here, to advance this emerging field, we suggest that perceptual changes and the increased pacification of action during defabrication may be plausibly explained in terms of computational mechanisms under AIF. We will in particular emphasize that a full account of defabrication must draw not only on perceptual inference, but its interplay with active inference in its technical sense of inference of policies for actions that will minimize uncertainty (Parr et al. 2022). This marks a crucial difference to predictive processing accounts grounded only in perceptual updating, which we argue fails to explicate key changes in action selection dynamics during meditative experience. Our account differs from previous accounts of meditative experience in this respect (e.g. Carhart-Harris and Friston 2019, Lutz et al. 2019, Laukkonen and Slagter 2021), as we will explain in the Section *Existing Work on Meditative Deconstruction under AIF.*

Methodologically, we enable a ‘translation’ of deep meditative phenomenology into computational phenomenology by synthesizing contemporary phenomenology with traditional Buddhist perspectives and frameworks (aligning broadly with Ramstead et al. 2022). We focus on the defabrication process undertaken by an advanced meditator in a formal session, assuming a pre-existing cultivated capacity to both perceive subtle mental tension, to release it and to fully defabricate experience.^4^

Overall, we develop a detailed account of how deep deconstruction plays out under AIF, including an illustrative example with associated phenomenology and plausibly implicated brain regions.^5^ We outline how deconstruction can culminate in the meditative endpoint of a ‘cessation event’, where all phenomenal experience ceases due to insufficient precision over even the lowest-level beliefs capable of sustaining conscious experience.

## Existing AIF work on meditative deconstruction

### Overview of AIF

AIF sets out how perception and action are plausibly intertwined and instantiated in the brain via neurocomputational mechanisms.^6^ According to the framework, the brain approximates Bayesian inference by instantiating a hierarchical generative model, which generates probabilistic hypotheses, or predictions, about the sensory input that those causes would give rise to, guided by a tractable objective function of minimizing variational or expected free energy (Parr et al. 2022, p. 25).^7^  *Perceptual inference* brings the model in line with sensory input, and *active inference* brings sensory input in line with the model. It is important to note that active inference specifically refers to inference of a *policy* for (mental or bodily) action, where the policy is a belief about state transitions given action which leads to expected observations (Parr et al. 2022). Calculating expected free energy for policies involves determining both their epistemic value, i.e. the expected information gain, and their pragmatic value, i.e. the probability of achieving preferred outcomes by following that policy. Active inference thus underwrites the explore-exploit distinction such that agents shift to explorative policies if policies expected to achieve pragmatic value have high expected free energy. It is these aspects of active inference we will bring to bear on defabrication in this paper.

The generative model is hierarchically structured, with higher-level beliefs encoding increasingly general and conceptually constructed beliefs about the sensorium, such that predictions and policies unfold over longer temporal scales, and lower levels correspondingly have predictions and policies unfolding over shorter temporal scales, which are less constructed (Limanowski and Friston 2020, Parr et al. 2022).^8^ Perceptual and active inference go together in a hierarchical sense with policies both entraining actions over longer scales higher up (e.g. episodic sequences) and action over shorter scales further down (e.g. sensorimotor sequences), following the brain’s control hierarchy (Pezzulo et al. 2018). Higher levels contextualize lower ones, which in turn provide evidence for them (e.g. seeing a best friend’s face contextualizes local facial features in a certain arrangement, with those facial features providing evidence that one is indeed looking at their best friend). These hierarchical aspects will be central in our account.

Finally, our account draws on the framework’s use of temporal depth and habits. Temporal depth reflects how far into the future actions are considered during policy selection at a given level of the hierarchy.^9^ The framework casts *habit formation* as a process where expected observations are used to form empirical priors. That is, context sensitive beliefs about how to act that influence policy selection independently of expected free energy relative to expected goal states (Parr et al. 2022, p. 93).

### Existing work on meditative deconstruction under AIF

There is an emerging literature which accounts for aspects of meditation in terms of AIF and more broadly predictive processing framings.^10^ Intriguing work includes explanations of focussed attention and dereification (Lutz et al. 2019), Zen practice (Pagnoni 2019), meta-awareness (Sandved-Smith et al. 2021), attenuation of maladaptive construal of body sensations (Farb et al. 2015), hypnosis and meditation states (Jamieson, 2016), mindfulness-based cognitive therapy (Manjaly and Iglesias 2020), meditation, concentration, insight and learning (Prest, 2021), and meditative selflessness (Deane et al. 2020). Pagnoni and Guareschi (2017) discuss predictive processes and Zen Buddhism, while Laukkonen and Slagter (2021) propose a unifying account of how meditation reduces temporal depth. Pagnoni and Guareschi (2021) propose a ‘work in regress’, a process by which agents reintroduce cognitive flexibility via meditation, unlearning or loosening beliefs in order to become more adaptive, with the mechanism being the relaxing of precision of ingrained beliefs to leave the agent more open to alternatives (*cf.* also Carhart-Harris and Friston 2019).

Our account partially aligns with work by Carhart-Harris and Friston (2019), Laukkonen and Slagter (2021), Lutz et al. (2019), while also drawing on notions from Pagnoni et al. (2021). We share the view of Carhart-Harris and Friston that meditation relaxes precision over high-level beliefs, with Laukkonen and Slagter that deconstruction decreases temporal depth, and with Lutz et al. (2019) and Pagnoni et al. (2021) that the process involves non-action.^11^ We now review these aspects in turn.

Regarding the relaxation of precision, we agree with the Carhart-Harris and Friston proposal that meditative states involve reduced precision over high-level beliefs about hidden states, driving perception to be determined by lower-level beliefs. This notion has been similarly expressed or adopted by Pagnoni and Guareschi (for Soto Zen meditation), and by Laukkonen and Slagter for open monitoring meditation. We argue that this computational mechanism is involved in all meditative deconstruction.

Regarding temporal depth, in the only existing work specifically targeting meditative deconstruction, Laukkonen and Slagter (2021) propose a ‘unifying account of deconstructive meditation’, that appeals to precision modulation such that ‘practising meditation [...] gradually reduces counterfactual temporally deep cognition, until all conceptual processing falls away’ (p. 199). The account presents the meditation techniques of focused attention, open awareness, and non-dual practice on a spectrum. As noted, we agree with the overall approach of appealing to precision modulation in accounts of phenomenology in general, and in particular for techniques marked by reducing temporally deep cognition. However, here, we move down from the meditation technique level to directly target the cognitive or computational process of defabrication itself. This focus is partly motivated by the observation that one technique will not necessarily reliably produce the phenomenal experience it aspires to, nor necessarily lead to the full gamut of meditative experience (Matko and Sedlmeier 2021, Yaden 2022, Lutz et al. 2015). Furthermore, in deeper states, technique is often reported as ‘dropping away’ (Lee Dhammadharo 1995, Maha Bua 2012). Hence, we suggest that it is the process of deconstruction itself, not just the technique, that provides a rich and nuanced level of analysis for developing an AIF account.

The notion of non-action plays a key role in predictive processing accounts of several different types of meditation by Pagnoni and Guareschi (2017) and Lutz et al. (2019), arguing for an active vigilant immobility as causal for the impact of Zen meditation, or for non-action as a mental action which sets high precision to sensory prediction errors specifically associated with an attentional target. We agree with these accounts that non-action plays a role in meditative deconstruction, yet focus on its role in deconstruction independent of technique, providing a distinct, nuanced account appealing to formal AIF for how non-action initially supports—yet is later increased—by deconstruction, which we will argue for in the Section *Deep Meditative Deconstruction under AIF*.

Our account importantly differs from the aforementioned work in terms of both phenomenology and computational mechanistic specificity.

First, our detailed synthesis of the phenomenology and process of defabrication permits us to put the release of mental tension into relation with other key notions. This enables us to computationally account for these notions, including equanimity, stillness, present-moment experience, cessations, and the role played by traversal of the jhānas and the insight knowledges in the defabrication process. Specifically, we argue that, if we cast the release of mental tension as reducing precision over all beliefs about hidden causes at a given level of the hierarchical generative model, and if this release is repeated by the meditator at each level down the hierarchy, then the computational mechanisms of AIF entrain a recapitulation of the phenomenology of defabrication.

Second, our account is unique in explicitly linking, in a causal chain, the release of mental tension to the scoring of expected free energy, revealing deconstruction as an active rather than passive process of precision control. We propose that defabrication involves a downward cascade of increasingly lower-level active belief sampling within the hierarchical generative model—reflected perceptually in decreased conceptual complexity and actively in decreased higher-level policy selection. This cascade follows from the relationship between releasing mental tension and decreasing precision over all beliefs about hidden causes (states) at a given hierarchical level. Crucially, this weakens preference-led utility-seeking policies at that level, which necessarily drives exploration-based policies that recruit information from the level below.^12^ We argue that the release of mental tension, when repeated ever lower in the hierarchy, progressively attenuates higher-level influence on perceptual content and action selection—and thereby captures the phenomenology of defabrication. This impact on action selection reduces hierarchical depth as we set out in Section *Release of Mental Tension as a Reduction in Belief Precision, Driving Active Selection of Lower-Level Foraging,* with temporal depth of policies reduced only as a consequence (see Section *Pacification of Action*). An upshot of the process is that it induces non-action.

Our approach thus sets itself apart by both its extensive use of synthesized evidence for defabrication, and the detailed AIF account of the cascading precision-weighting mechanism involved, appealing to the formal process by which policy selection occurs under AIF via the weighting of pragmatic and epistemic value in scoring expected free energy.

### AIF and phenomenal experience

It is conscious, phenomenal experience that is being defabricated in the meditative practices we focus on. Under AIF, phenomenal experience is associated with the system’s posterior belief after engaging in inference, and is thus the upshot of an otherwise unconscious process (Hohwy 2013, p. 201; Friston 2013, p. 39; Seth and Friston 2016, p. 5; Whyte et al. 2026).^13^ We can elucidate this idea, including the role of active inference, with recent explanations of binocular rivalry that link dynamic processes across the hierarchy to phenomenal experience, with particular reference to meditators’ altered experience of binocular rivalry.

In binocular rivalry, each eye is presented with a different image and perception alternates between them rather than merging them; when presented with an image of a car and a house, participants alternate between seeing one or the other, with brief periods of mixed percepts in between. Predictive processing accounts explain rivalry in terms of competing high-level beliefs about hidden causes that do not overlap spatially (Hohwy et al. 2008, Weilnhammer et al. 2017). Findings indicate that long-term meditators have altered perception in rivalry. A study on Buddhist monks (Carter et al. 2005) found that the otherwise brief transition or ‘mixed percept’ periods (mixed patches from each image) were prolonged in one monk, which Laukkonen and Slagter (2021) interpret as evidence of weakened high-level beliefs about non-overlapping hidden causes. Another study found that meditation increases mixed percepts in long-term meditators, proposing this being due to disabling the communication of bottom-up sensory information to higher regions (Katyal and Goldin 2021, p. 21), which can be understood as attenuating higher-level competition among beliefs, as if ‘pushing’ perceptual inference down to more local, transient sensory attributes (see also Prest 2021, p. 41).

**Figure 1:**
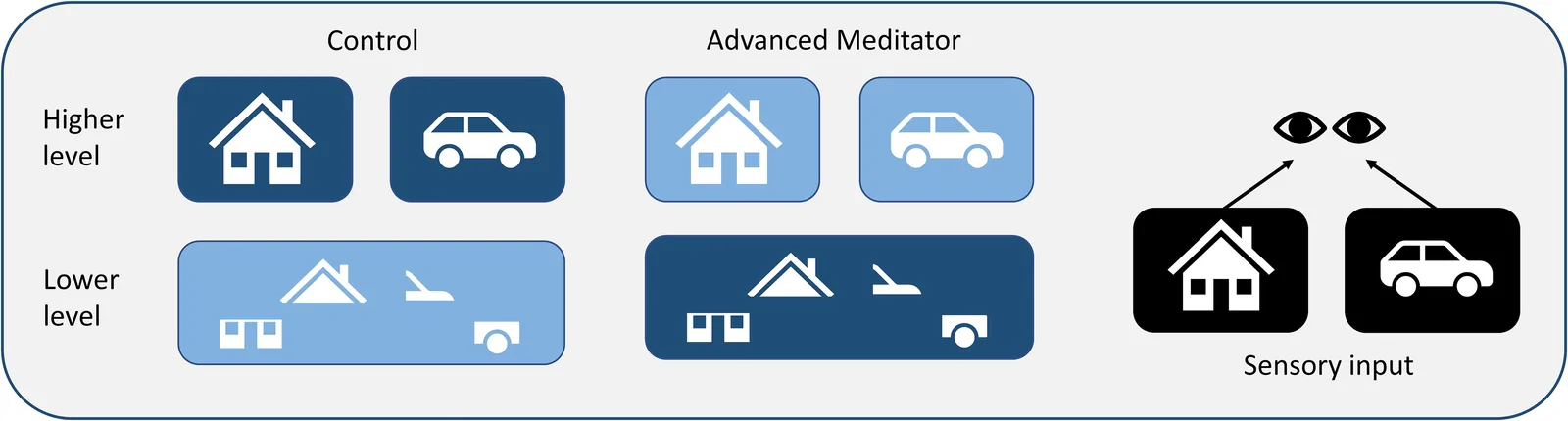
Phenomenal experience and hierarchical processing of meditators under binocular rivalry. Control group on left and advanced-meditator group on right. Dark blue boxes represent beliefs featured a higher proportion of the time in phenomenal experience. Light blue boxes represent beliefs featured a lower proportion of the time. The advanced meditator group has decreased proportion of L2 beliefs leading to more time perceiving lower-level mixed percepts rather than exclusively ‘house’ or ‘car’.

Active inference is involved in this process, as recognizing a visual object involves saccades around the image and/or shifts in covert spatial attention to confirm the predicted patterns consistent with perceiving that object (Slotnick and Yantis, 2005, Friston et al. 2012, Mirza et al. 2016, Parr et al. 2019). Saccades and endogenous feature attention seek to confirm features like lines, shape, texture and so on, and their configuration. When perceiving mixed percepts, the eyes no longer perform saccades or direct attention to confirm the current higher-level hypothesis—‘I am perceiving a car’ need not be confirmed. Instead, active inference engages to confirm lower-level hypotheses, such as ‘I am perceiving a vertical light-dark contrast’ (see Fig. 1).

It is this kind of understanding of phenomenal experience that we will bring to bear on defabrication. Before giving our account, we need to unpack Buddhist meditation-induced defabrication and its phenomenology.

## Defabrication under a contemporary Buddhist phenomenology

For a balanced yet nuanced understanding of Buddhist defabrication, we combine contemporary phenomenological (e.g. Boowa 2012, Burbea 2014) and traditional sources (e.g. Nagarjuna, Pali canon). We argue this is the best approach, permitting phenomenological accounts from advanced meditation practitioners (e.g. Boowa 2012, Pandita and Aggacitta 2012, Burbea 2014, Sayadaw 2016), while admitting supporting traditional frameworks. While Buddhist scholarship admits competing interpretations, our approach avoids both rigid or debatable parochial interpretations of traditional accounts and overreliance on potentially misleading anecdotal, idiosyncratic contemporary phenomenological reports, and interpretation. Rather, it seeks common ground between established Buddhist frameworks and phenomenological accounts by advanced meditators who claim to experientially comprehend them.^14^ More specifically, we use ‘advanced meditator’ here to refer to practitioners who claim to experientially comprehend the contemplative terrain analysed in this section. The aim is a phenomenologically grounded account of the construction of experience which can subsequently be empirically substantiated; this matters for the AIF account we will present because the picture of defabrication that emerges is more staged, active, and dynamic than might be otherwise supposed.

### Defabrication: endpoints and phenomenology

#### Cessations

Buddhist defabrication comes under many guises and can also be referred to as the fading of perception (Burbea 2014, Chapter 19), the pacification of all objectification (Nagarjuna 1995, v. XXV:24), or the unbinding of conditioned experience (Bodhi 2012; AN 9.34). Defabrication lies along a continuum, with the greatest degree of defabrication in Buddhism being extinguishment (*nibbāna*, liberation), marked by no fabrication or mental tension, and the greatest degree of fabrication being the depths of extreme suffering (*dukkha*), marked by high fabrication and mental tension. Extinguishment can take the form of a cessation of consciousness, described below.

Before proceeding, we disambiguate the term ‘cessation’, a key reference point in the defabrication process. While interpretations of cessations vary significantly across various practitioners or lineages,^15^ we employ the term *cessation event* to refer to a momentary full cessation of phenomenal experience ‘after the full traversal’ of the samatha jhānas (concentrative meditative absorptions) or insight knowledges (*vipassana-ñanas*).^16^ This state is not conscious, yet there is a subsequent awareness that it occurred (Bodhi 2016, pp. 358, 363; Sayadaw 2016, pp. 445–446; Berkovich-Ohana 2017; Chowdhury et al. 2023).

In traditional sources, this type of cessation event is described as ‘supramundane absorption with Nibbāna as object’ (Bodhi 2016), that can occur once an advanced meditator has previously achieved ‘fruition’ or one of the stages of awakening (*ariya-phala*; Thera 1979). Such events represent a temporary endpoint to the fabrication process. As Burbea (2014) notes:

At the extremity of the more subtle end of the continuum, in a moment when clinging, identification, and grasping at inherent existence are pacified, there is the cessation of perception [. . .] [T]he cessation of perception comes about through a dying down of fabricating. (pp. 229–230).

Therefore, a meditator can understand that they have fully defabricated phenomenal experience by entering the non-state of a cessation event.^17^

#### Equanimity and stillness

Defabrication involves the progressive reduction of clinging as one releases mental tension, and with less clinging, there is a reduction in perceptual construction. This leads to increased *stillness* (*passaddhi*) in experience due to increased *equanimity*, the capacity to remain non-reactive yet open to what arises in experience. Burbea (2014) notes the relationship as follows:

[E]quanimity—the dying down of the mind’s push and pull with respect to phenomena—does not reveal a world of things, in their pristine ‘bare actuality’, ‘just as they are’, stripped only of the distortions of ego projections [. . .] Rather, as meditative skill develops, we see that without clinging phenomena do not appear at all. Not only how they appear, but that they appear is dependent on the fabricating conditions of clinging. (p. 299).

That is, perceiving with more equanimity is perceiving with less fabrication in experience. Equanimity changes what we perceive rather than providing us with access to ‘the way things really are’. Therefore, one phenomenological marker in the AIF account we will propose is that the degree of equanimity also correlates with the degree of defabrication.

### Phenomenological variation within defabrication

We adopt an approach where, at each stage of defabrication as one releases mental tension and moves toward a cessation event, the moment-by-moment quality of experience can vary along a spectrum (Pandita and Aggacitta 2012, Burbea 2014). At one end, experience features very little flux of sensations, more uniform content, less investigation, and higher affective valence. At the other, there is higher flux and valence, more varied content and more investigation. The former are commonly referred to as the samatha jhānas, and the latter, the insight knowledges. The jhānas are a set of tranquil meditative absorption states (Burbea 2014, Ṭhānissaro 2006, Polak 2011, Arbel 2017), while the insight knowledges are characterized by changes in cognition, perception, emotion, feelings, and consciousness (Buddhaghosa 2020, Sayadaw 2016, Chowdhury et al. 2023).^18^

In what follows we provide a characterization of the step-wise phenomenology of defabrication until its culmination in a cessation event, focussing primarily on the jhānas as their higher degree of uniformity allows for clearer presentation of how decreased abstraction, increased equanimity, increased stillness, and more pleasant valence are progressively attained. We also summarize defabrication via the insight knowledges to show that the commonly employed insight path to cessation is a distinct version of the same step-wise process.^19^ Including both sides of defabrication supports a more inclusive portrayal grounded in Buddhist literature and clarifies that our account of defabrication seeks to explicate the underlying common computational mechanism driving transitions between one stage and the next to attain construct validity, rather than accounting for all experiential aspects for high face validity. This ensures that defabrication is not conflated with any single ‘vehicle’ by which it is achieved, but is understood as a more generalized step-wise computational process underlying any particular way meditators defabricate.

### Progressive defabrication to cessation

What does this progressive defabrication via the jhānas to a cessation event look like in a single meditation session, which is our focus in this paper? Burbea (2014) suggests that states such as the jhānas always feature some degree of letting go and reduced craving, involving progressively less fabrication of perception (p. 305). With each stage, stillness and equanimity deepen accordingly, while valence becomes more positive.

As the meditator focuses on a central object of mindfulness (*parimukham*) with greater attentiveness (*vitarka*) and investigation (*vicāra*), they withdraw (*vivicceva/vivicca*) from sensory disturbances, conceptual proliferation (*papañca*), and hindrances (*nīvaraṇa*). This unbinding process gradually stills perception. As mental tension diminishes, rapturous (*pīti*) and non-sensual pleasant states (*sukha*) arise, which feel lighter and more ‘open’. This initial fading of perception marks the first jhāna (Ñāṇamoli 1995; MN 43), where a nascent sense of stillness begins to permeate awareness.

Further release of coarser fabrications gives rise to the second jhāna, characterized by more effortless calm (*passaddhi*), inner tranquility (*sampasadana*), and unified awareness (*ekaggatā*). These reflect reduced top-down attentional deployment, increased stillness and a reduction in mind-wandering, with awareness becoming more stabilized. Here, contracted tensing to any sensual experience starts to drop away, leaving experience more spacious. The emerging stillness becomes steadier, supported by a subtle undercurrent of equanimity. In the third jhāna, the subtle fabricated tension of rapture of the second jhāna is released, giving rise to meditative equanimity (*upekkhā*), increased satisfaction and serenity, with discursive thought rarely appearing (Yates et al. 2017). In the fourth jhāna, even subtle pleasant states and discontent dissolve, leaving only deepened equanimity (*upekkhāsatipārisuddhi*), a sense of impartiality, with freedom from both pleasure and pain (*adukkhamasukham*). Sensations of breathing are fully absent (Thanissaro 2008), with a state of refined contentment pervading awareness (Bodhi 2012: AN 5.28; Bodhi 2016) containing deep fulfilment and satisfaction (Dhp 110; Dhp 372–74), where equanimity and stillness now predominate.

Even these states come to be perceived as contracted (Bodhi 2012; AN 9.42; Ñāṇamoli 1995; MN111), indicating that aspects of experience have not yet fully ceased, as refined mindful equanimity persists in the perception of form. Form (*rūpasaññānaṁ*) refers to phenomena, which are attributable to external sources (i.e. the five senses) or the body, whereas ‘formless’ refers to phenomena not perceived as originating from the external world via the five senses or the body. Further defabrication moves the meditator into the formless jhānas (*arūpa-jhānas*), states characterized by an absence of form.

As perceptions of form cease, the meditator releases perceptions of phenomenal variety (*nānattasaññānaṁ*) and bounded (sensory) resistance (*paṭighasaññānaṁ*), entering the fifth jhāna of boundless space (*ākāsānañcāyatana*). Phenomenologically, this feels like localized experience dissolving and expanding beyond the body’s usual boundaries, as the sense of having a body or form has vanished, with unhindered stillness pervading. However, when this state of boundless space begins to feel confining, the meditator can release into the sixth jhāna, boundlessness of consciousness (*viññāṇañcāyatana*)—described as consciousness and presence pervading the field of experience (Ingram 2018, pp. 173–174). Here, stillness is no longer felt as a quality within experience but as the very atmosphere of awareness itself, and equanimity is implicit in the absence of resistance or differentiation. As release continues and boundless consciousness feels too contracted, further defabrication leads to the seventh jhāna—dimension of nothingness (*ākiñcaññāyatana*) where there is no longer a perception of space (Burbea 2014, p. 305). The sense of spatial orientation, previously devoid of sensory objects in the fifth jhāna, is now absent. Yates et al. (2017) describe seventh jhāna as the only meditative mental construct represented by an absence rather than a presence. Stillness here is unmarked and diffuse, becoming a quiet backdrop to the vanishing of perceived structures.

Perceiving ‘nothingness’ in the seventh jhāna still involves a subtle construction of the experiencer, producing very subtle constriction. Releasing this allows ‘nothingness’ to cease, leading to the eighth jhāna of neither perception nor non-perception (*nevasaññānāsaññāyatana*), where experience is reported to disappear while still remaining a phenomenal state. Phenomenologically, there is a stillness no longer experienced as a quality, but simply reflected in the absence of any tension or fabrication. Although this might seem like an endpoint, further defabrication is possible through a cessation event.

In contrast to traversal to a cessation event via the jhānas, the insight traversal involves investigation into specific aspects of experience, in which perception is defabricated by adopting an ‘insight way of looking’. This involves investigating the three characteristics: impermanence, suffering and non-self which reduces mental tension and clinging (Burbea 2014, Payutto and Prayut 1995, pp. 101–157). In this context, the insight knowledges characterize the stages of progression to cessation events. To reach a cessation event *via* the insight knowledges an advanced meditator must develop and move through them starting with the ‘knowledge of arising and passing away’, passing through the knowledges of ‘dissolution’, ‘fear’, ‘misery’, ‘disgust’, ‘desire for deliverance’, and ‘re-observation’ until the ‘knowledge of equanimity’ (Buddhaghosa 2020, pp. 732–733).^20^ When ‘knowledge of equanimity’ is mature, one moves to ‘knowledge in conformity with truth’ (*saccānulomikañāṇa*) followed by ‘knowledge of change-of-lineage’ (*gotrabhūñāṇa*), and finally to the cessation event,^21^ appears to involve similar phenomenology to the insight route, with experience characterized by flux, and progressively moving to more equanimous and positively valenced, yet less fabricated, experience.).

#### Summary

We have characterized defabrication via the jhāna side of the spectrum, with brief reference to the insight side. This serves to illustrate how defabrication may be accomplished with varied techniques and phenomenology. An advanced meditator, by traversing along either side—or an intermediate point marked by partial flux, limited phenomenal variety, and intermediate valence—progressively defabricates to a cessation event. Despite these differences, in all cases the core underlying change is the progressive release of mental tension, reducing fabrication with each progressive step, from one jhāna or insight knowledge to the next, with the computational mechanism being a central focus of our account. Our synthesis of the relationship between clinging, mental tension, valence, equanimity, decreased action, cessation events, the jhānas and the insight knowledges in advanced meditation establishes the necessary relational connections for our AIF deconstruction account.

## Deep meditative deconstruction under AIF

We now investigate how mental tension may plausibly function in terms of AIF mechanisms, providing an account of how the Buddhist defabrication process, as enacted by an advanced meditator in a given session, deconstructs experience.

### Release of mental tension as a reduction in belief precision, driving active selection of lower-level foraging

The starting point for this account is the meditator—hereafter called ‘the system’—having achieved an on-demand capability to perceive subtle mental tension.^22^ Perception of such mental tension necessarily means the establishment of beliefs about whether or not, and to what extent, mental tension is being experienced at a given moment (recall the example in the Introduction of the meditator perceiving a visual object and noticing a dense pressure behind the eyes). These beliefs form a part of the system’s best explanation for current sensory input. As all perceptual content is understood to come with associated mental tension, the system can target the adaptive disengagement from the inference process at a given level of the hierarchy by stopping the associated tensing via a mental action.^23^ Such an action is akin to an attentional act, in the sense that it alters the contents of perception, yet is not attentional in the sense that it targets a particular object to attend to.

We propose that experientially, it is the release of mental tension at a given level which correlates specifically with decreased precision over all beliefs about hidden causes (that is, about the state of the world and the organism itself) at that level.^24^ It is by combining this starting point with the AIF mechanisms of action selection, and release repeated at each level of the hierarchy, that defabrication and its phenomenology can be accounted for and explained within the framework.

An immediate consequence of reduced posterior belief precision over hidden states at a given level of the hierarchy is the impact on action policy selection. With AIF, the decreased confidence in beliefs means that pragmatic value seeking actions based on our current, uncertain knowledge become risky, since we can end up with outcomes we wish to avoid rather than the ones we wish to attain.^25^ That is, if we are uncertain of the outcome of our actions, it is better to learn more before acting. Furthermore, the more the uncertainty the more opportunity there is to learn, which upweights policy selection for epistemic value. This tilts action in favour of a kind of exploration or epistemic foraging due to the way expected free energy balances epistemic and pragmatic action selection, as mentioned in Section *Overview of AIF.*

Under normal circumstances, epistemic foraging could potentially entrain a degree of gross action, with the agent moving about to learn more (e.g. moving closer to and picking up an object to get a closer look). However, two constraints prevent this in the case of meditative deconstruction. The first is that the decrease in certainty over all beliefs at that level, rather than a particular belief, means that largely epistemic actions which entail expected pragmatic risks are not taken—if all my possible movements have unknown consequences, it is best not to move at all lest I step off a cliff. This constrains the kind of action taken to epistemic actions without pragmatic consequences, which are those which forage, without gross movement, at the level below. The second constraint on gross action is the impact of meditative training on contextual habits. When a meditator sits down on the cushion, their repetition-established habits impact policy selection irrespective of free energy minimization, and make policy selection involving movement less likely (i.e. meditators have a habit of not engaging in gross bodily movement). These two constraints leave the system with only one good option, attempting to resolve insufficient precision by engaging in lower-level exploration, thereby selecting policies which entrain actions that resample at the hierarchical level below with the aim to re-establish certainty about the higher-level belief (Parr and Friston 2017, Pezzulo et al. 2018). Such low-level foraging has been previously modelled (Friston et al. 2017, Heins et al. 2020, Smith et al. 2022, p. 40), where, if there is sufficient uncertainty about what is being perceived, based on prior beliefs about higher-level states, a policy is selected to entrain sensory organs to the predicted best lower-level location to resolve uncertainty. In sum, lower-level exploration due to its lack of pragmatic risk and potential for information gain is the best path to expected free energy minimization under the circumstances of a level-wide reduction in belief precision, and this policy selection tendency is reinforced through habits that further downweight policies for gross bodily action.

**Figure 2:**
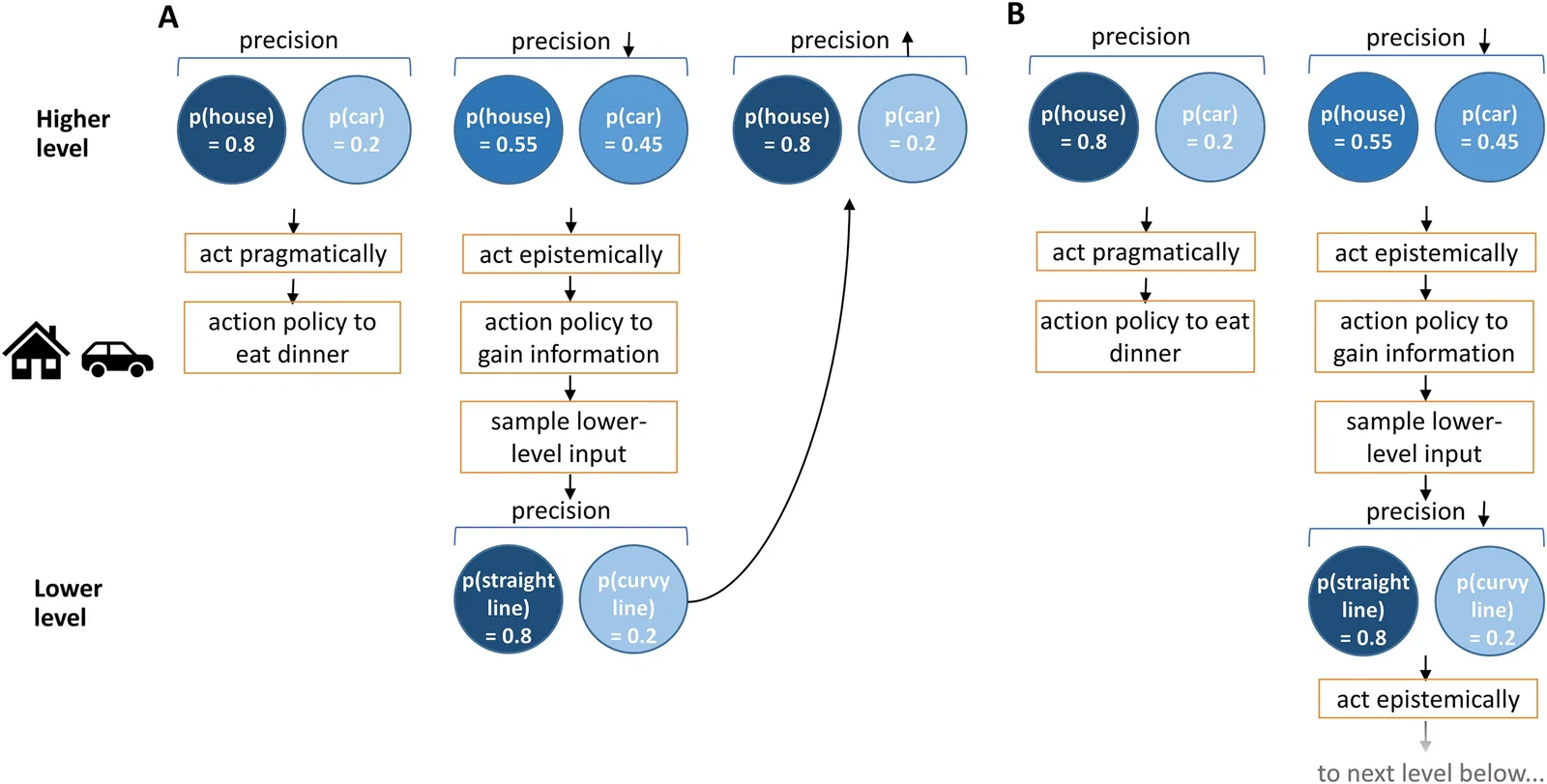
Lower-level inference via decreased higher-level belief precision. Two higher-level beliefs that the system is perceiving a car or a house (left column of panels A and B) in a probability distribution. Decreasing the precision over that distribution (middle column of panels A and B) decreases certainty about perceiving a car or a house. This tilts the system toward favouring epistemic over pragmatic value pursuit, with an epistemic policy being selected to gain more information which results in inference at the lower level (in this case about the shape of the perceived object). In panel (A), once lower-level inference is completed, confidence about higher-level beliefs can be re-established (right column of panel A). In panel (B), the system repeats the precision reduction at the lower level via the release of mental tension, resulting in epistemic action to move down to an even lower level of inference (right column of panel B).

As discussed earlier regarding binocular rivalry, meditators experience more ‘mixed percepts’ during rivalry, which can be interpreted as moving perception down to a lower level, to where there is fluctuating and milder rivalry between more local sensory attributes. We suggest that a similar downward move happens in normal, non-rivalry perception, with meditators’ more defabricated experience featuring less higher-level conceptual representation. If sensory input is explained higher up the hierarchy, then experience involves more fabricated and conceptually constructed content. In the car/house rivalry example, this was at a level where inference of ‘house’ or ‘car’ could be identified by the system. When it is too risky to act at the level of the concepts car/house, and there is increased potential to gain information, the system selects an epistemic policy entraining lower-level inference, with experience now reflecting beliefs encoded at the lower level, closer to sensory input, which now provide the best explanation for that sensory input. That is, experience has moved to a level below concepts such as ‘house’ or ‘car’, since no attempt is made to integrate them conceptually. In this way, meditators’ perception in binocular rivalry provides evidential support for our proposed mechanism, illustrating how meditators can move down perceptual levels (see Fig. 2, Panel A).

Meditator-initiated defabrication can therefore be understood as a process underlying the movement of perceptual inference to lower-levels where the posterior beliefs associated with phenomenal experience, such as ‘mixed percepts’ and other lower-level sensory attributes, are encoded. This movement to lower-level inference occurs via epistemic action due to the comparatively lower expected free energy of epistemic policies. That is, defabrication drives perceptual experience down to levels where more abstract concepts can no longer be identified by the system. This reflects a reduction in the conceptual complexity or degree of abstraction of phenomenal experience (e.g. experience changing from perceiving the concept ‘car’ to perceiving some feature of the car such as its shape), yet importantly for deep deconstruction, the process must repeat at each level of inference.

Under non-meditative conditions, having hierarchically moved one level lower, the system would then successfully resample via inference at that level, with this followed by a subsequent move back up to then re-establish higher-level belief precision again in order to reinitiate the pursuit of pragmatic value (see Fig. 2, Panel A). Crucially, this is why in meditation the same move to target the release of mental tension must be repeated again at the lower level to instead drive inference even lower. Under the conditions of meditative deconstruction, instead of moving back up, beliefs at the lower level are *also* rendered increasingly imprecise, attenuating pragmatic policies at that level in favour of even lower-level exploration (see Fig. 2, Panel B). In this way, the system moves epistemic foraging ever-lower in the hierarchy as the probability of pragmatic policy selection decreases at each subsequent level due to the ever-increasing relative weight of epistemic policies. This results in reduced (functional) hierarchical depth. It is this kind of mechanism that underwrites each of the steps of the jhānas or insight knowledges, with the phenomenological changes with each downward step reflecting the lower hierarchical level at which the experiential content is encoded. This occurs over all stages until the cessation event, when the inference finally drops to a level where posterior beliefs are no longer associated with conscious experience.

### Pacification of action

**Figure 3:**
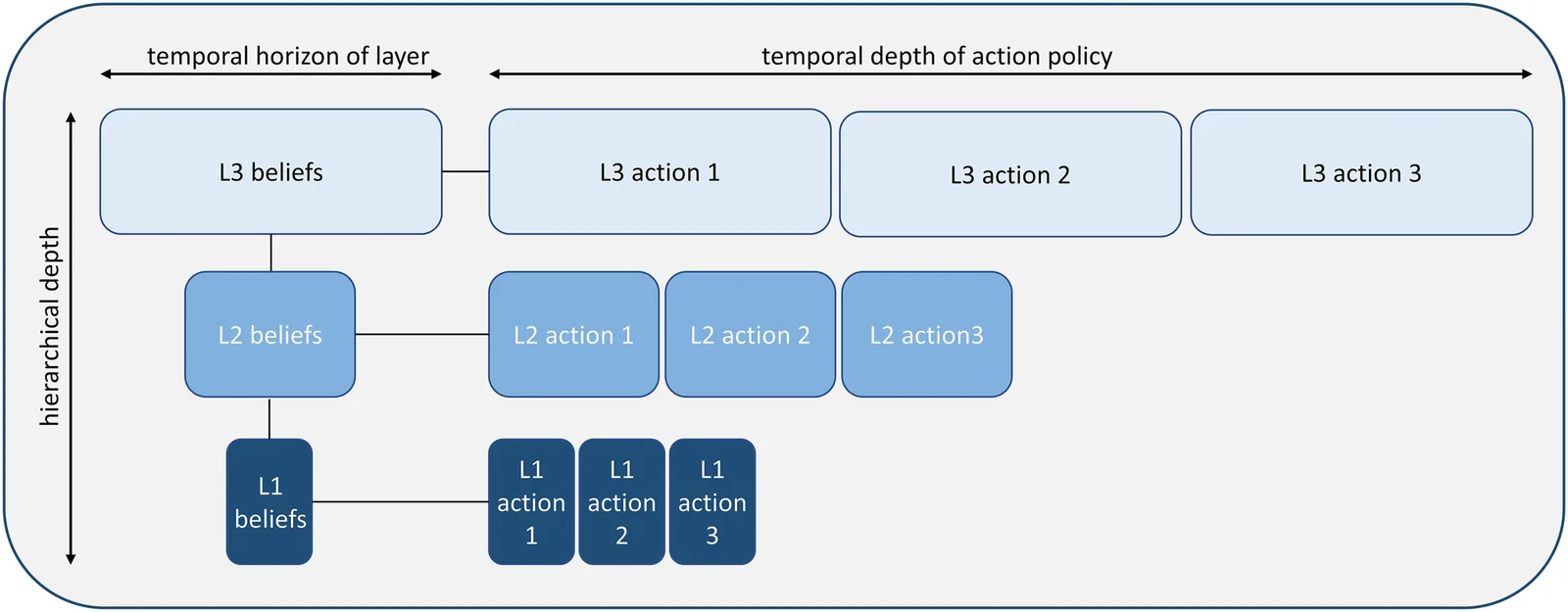
Decreased temporal depth during meditative deconstruction. Reducing precision on Layer 3 (L3) conceptual beliefs, represented in phenomenal experience, undermines policies at that level of hierarchical depth. Here, L3 beliefs have been rendered too uncertain to provoke L3 policy selection. Layer 2 (L2) beliefs are more imprecise than normal, yet still occasionally become certain enough to trigger L2 policy selection. Layer 1 (L1) beliefs do not have compromised precision, and thus continue to trigger L1 policy selection as they normally would. Notice that L1 actions occur over a shorter temporal horizon and thus have less temporal depth when compared to L3 actions.

A key phenomenological quality of deep meditative deconstruction is, as mentioned above, the increased experience of stillness, which deepens as the meditator progresses through the jhānas or insight knowledges, for example becoming very prominent by the fourth jhāna. We argue that this experiential quality reflects the decreasing selection of higher-level pragmatic policies. We have already discussed how the repeated release of mental tension decreases belief precision, reducing pragmatic policy selection and increasing epistemic policy selection, thus driving inference to progressively lower levels in a step-wise fashion. According to AIF, and mentioned in Section *Overview of AIF*, generative models are hierarchically structured such that when the system moves to a lower level of the hierarchy, the temporal duration of each action in the policy decreases. This is because the length of an action at a given level is determined by the timescale of the regularities tracked at that hierarchical level (for example, 100 ms). Therefore, policies will tend to occur over shorter timescales as the system moves down the hierarchy, that is, their temporal depth is reduced (see Fig. 3). Thus, as the system moves down to lower levels, the policies occurring over the longer temporal scales of the levels above are no longer selected, shortening the time span of policies, entailing a reduction in longer and more complex actions. In the car/house example from Fig. 2, the sequences of actions entrained by a confident identification of a car, encoded higher up the hierarchy, occur over a longer temporal horizon than actions entrained by the confident identification of less processed perceptual elements encoded lower in the hierarchy, such as visual patches, edges, or lines. In both cases, moving from the higher to the lower level reduces hierarchical depth, which has the consequence of reducing the temporal depth of policies (i.e. their duration), thereby restricting the action space to subtler and less elaborate potential actions (Fig. 3).^26^ This restriction applies to mental actions as well, with the temporal duration of processes such as rumination being reduced. Therefore, the experience of increased ‘stillness’ or ‘pacification’ of the mind and body in deconstruction is ultimately a consequence of the repeated release of mental tension.

To give an example, consider that sustained thoughts about one’s self in relation to others are underpinned by high-levels encoding beliefs about ‘oneself’ and ‘others’. The system must be running inference high up the hierarchy for such beliefs to be reflected as conceptual understanding in experience (*cf.* ‘what I want’ and ‘what they want’). Thus, the meditator defabricates and starts constraining experience to the more limited self and other beliefs encoded at lower levels of the hierarchy. The insufficient higher-level belief precision means they avoid selecting and enacting policies in response to goals pertaining to the high-level self and other beliefs since they are too risky. For advanced meditators, due to repeated practice deconstructing (see habits in Section *Release of Mental Tension as a Reduction in Belief Precision, Driving Active Selection of Lower-Level Foraging*), such avoided policies will tend to already consist predominantly of mental rather than bodily actions, and are likely to be phenomenally experienced as grasping or aversive thoughts (ones in pursuit of pragmatic value), reflected in activities such as mind wandering.

An area of potential concern here is how the system pursues a sustained meditative policy (i.e. deconstructing through all the jhānas to a cessation event over a single meditation session), entailing repeated mental tension release step-wise down the hierarchy, given that high-level pragmatic value pursuit is attenuated. This could be thought to inhibit the pursuit of the necessary policy seeking the preferred outcome of the cessation event when beliefs become too imprecise at the level such policies operate at. The response to this concern is that the deconstruction process, even over a single session, is not linear. On average, higher-level inference takes place proportionally less and less of the time, yet still intermittently occurs far into the process. For example, during the first jhāna narrative thought is reduced but can still occasionally arise. This means that the high-level meditative policy (involving what is necessary to traverse the jhānas or the insight knowledges) is revisited, though more infrequently due to progressively less precision on high-level beliefs. Furthermore, the advanced meditator’s strongly developed habits of releasing tension mean that the process is likely to be carried out due to, through habitual repetition, the establishment of empirical priors at each level of the hierarchy. These empirical priors are influenced by the level above, and influence policy selection independently of current expected free energy (see Parr et al. 2022, p. 93 and Smith et al. 2022, p. 41). This proposal is consistent with the finding that acquired habitual skilled behaviour does not require cognitive control after learning has occurred (Bassett et al*.* 2015). The upshot of all this is an automated tendency to reflexively release mental tension.^27^ This is particularly crucial in the late stages of deconstruction when higher-level inference is fully offline. The ‘forage lower’ policy selection is carried through to a cessation event, as we will describe in Section *Illustrative Example of Full Deconstruction*, based on this kind of cultivated habitual inertia.

### Equanimity and valence

We noted in Section *Defabrication: Endpoints and Phenomenology* that the degree of equanimity correlates with the degree of defabrication. Having examined the effects of decreasing belief precision on policy selection, we are equipped to discuss equanimity mechanistically.

Equanimity increases as higher-level pragmatic policy selection is curtailed due to the process described in the previous section. As we have discussed, pragmatic policies are those which pursue the system’s preferences. When sensory input is presented to the system, those high-level policies drive the agent to respond to that input in the ways which benefit the organism. In a sufficiently safe environment, such as that in which the meditator chooses to deconstruct, decreasing the selection of these high-level policies reduces the more conceptually driven responsiveness to phenomena. Non-reactivity is thus the consequence of high-level preference seeking policy attenuation. For example, a meditator moving from ‘third’ to ‘fourth jhāna’, or from ‘knowledge of arising and passing away’ to ‘dissolution’, increases their equanimity—thereby reacting less—because the inference process is more constrained to lower-level policies, with less abstract beliefs, restricting preference seeking behaviours to more limited, utility-irrelevant temporal horizons.

The system spending proportionally more time lower in the hierarchy has not only increased equanimity, but should also, according to the meditative view, have increased positive affective valence. Our argument for more positive valence lower down the hierarchy takes the following form: if the phenomenological changes induced by defabrication—of decreased conceptual complexity and increased equanimity, stillness and more present moment (shorter temporal scale) perception are due to perception moving down the hierarchy as we have argued, then since these phenomenological changes all correlate with increased positive valence, valence changes in deconstruction appear to be a hierarchical phenomenon as well. This proposal makes intuitive sense because the free energy is equal to complexity minus accuracy (Parr et al. 2022). While the more common accounts such as Hesp et al. (2021) associate valence with increasing accuracy. Here, we argue for the other side, consistent with the cognitive effort literature, that in the case of deconstruction it is the reduced complexity of decreased hierarchical depth which results in the more positive valence (Pezzulo et al. 2018, Vogel et al. 2020). The meditator can therefore be conceived as having cultivated a way to increase positive valence by, in a transitory period of time, reducing the hierarchical complexity of their model. That is, meditative defabrication could be cast as one way of reducing the negative valence of model complexity.

A possible objection to our argument about decreases in hierarchical depth being less computationally costly is that lower-level inference—due to those lower levels encoding many unconstrained fine-grained fast-updating beliefs about the world rather than higher-level summary beliefs regarding that world—would actually be more complex. Lower levels are indeed more complex in this sense. However, the higher levels are more computationally complex not only in terms of the multitude of abstract beliefs maintained over extended temporal scales (corresponding to ongoing long temporal scale regularity tracking), but more crucially due to the extensive counterfactual processing involved in planning and action policy selection at those levels in pursuit of organism-level preferences. Organism-level decision making requires extensive calculation of possible futures through expected free energy minimization in order to limit survival-related risk. The higher in the hierarchy processing occurs, the further into the future the agent must consider. As such, we argue that these higher-level computational complexity costs end up far outweighing the lower-level complexity costs. This argument can be assessed through hierarchical computational modelling of an active inference agent; we note it is consistent with empirical evidence of the higher metabolic cost of the upper levels of the cortical hierarchy (Jamadar et al. 2025). This would mean that suspending lower levels and only running higher levels would not result in the same computational cost savings as suspending higher levels.

### Illustrative example of full deconstruction

We can now illustrate progressive meditative deconstruction over a single meditation session under AIF, moving downward through hierarchical perception and passing through one particular sense modality, visual perception. The example here could be read as a case where the meditator starts the session with eyes open, which is not uncommon in meditation (Wallace 1999). Our illustration starts by reflecting general perceptual trends (with perception understood broadly and including the associated experience of thoughts), situating key insight knowledges or jhānas along the traversal toward the cessation event. We also highlight relevant neuroscience findings associated with these phases of defabrication.

As discussed in Section *Deep Meditative Deconstruction under AIF*, the system (meditator) initiates deconstruction by beginning to meditate according to their chosen technique for entering the jhānas or to begin insight investigation. This corresponds to starting in the ‘insight knowledge of arising and passing away’. Prior to visual deconstruction, the process initially most strongly influences higher levels in the hierarchy, associated with self-narrative related thinking, with the default-mode network (DMN) implicated (Friston 2018, Friston 2017, Carhart-Harris and Friston 2019). The DMN has been shown to be suppressed under meditation, reflecting meditation-driven attenuation of these higher levels (Zagkas et al. 2023).

Precision is reduced over beliefs regarding self-narrative content and related thoughts by the release of mental tension, moving experience and the inferential process hierarchically downward, resulting in proportionally less inference concerning self-narrative beliefs on average over time, as lower-level information seeking is increasingly favoured over self-narrative driven policy selection (reflected experientially as less self-narrative related thoughts arising). This continues until such content is either entirely or largely absent from the mix of phenomenal experience.

The system gradually moves down to inference concerning sensory integration, where the same process re-occurs with lower-level conceptual beliefs regarding objects, such as conceptual labels for objects (reflected in experience as less high-level conceptual recognition), where the superior temporal sulcus and middle temporal gyrus have been implicated; evidence suggests this region to also be suppressed under meditation (Farb et al. 2010, Beauchamp et al. 2004). Again, reduced belief precision at this level tilts policy selection toward epistemic over pragmatic policy pursuit. Note that progressive belief precision reduction at one level may temporally overlap with the same process at the next, yet on average precision is reduced to a greater degree higher up. The system does not necessarily disengage fully from pragmatic selection at one level prior to increasingly disengaging at the level below.

Next, in the visual system, visual experience is increasingly deconstructed, with decreasing precision on beliefs concerning viewpoint-invariant visual objects, implicating the with the lateral occipital complex (Vinberg and Grill-Spector 2008, Appelbaum et al. 2006, Grill-Spector et al. 2001, Kourtzi and Kanwisher 2001). Further release of mental tension moves the system down to now decrease precision on view-dependent objects, where the inferotemporal cortex has been implicated (Peissig and Tarr 2007). This is reflected experientially as a reduced conceptual complexity of objects, and consequently less selection of more conceptually driven policies. At this point, any policies underpinned by beliefs about what object the system is perceiving are increasingly less likely to be selected (such as the perception of concepts such as ‘car’ or ‘house’ mentioned earlier).

This degree of deconstruction in the meditation session, from the attenuation of viewpoint-invariant and dependent object beliefs, results in increased predominance of lower-level inference. This level features beliefs regarding basic form or shape and lines, such as those instantiated by the early visual cortex, which become increasingly prevalent in experience as higher-level influence on perception is attenuated. Studies on meditators support this, with a reported greater experiential prominence of aspects such as shape, size, three-dimensional depth and a loss of object boundaries in the visual field (Deikman 1963, Ataria 2015) in addition to pixelation, visual snow, and flickering (Lindahl et al. 2014). Loss of object boundaries is roughly analogous to reports of decreased body boundary perception in the ‘insight knowledge of dissolution’ (see Sayadaw, p. 21; Folk 2013, Sec. Dukkha Nanas). The system is now increasingly constrained to policies unfolding over very short temporal horizons that involve only mental actions on shape, object boundaries, etc*.* That is, for the meditator, experience has moved predominantly to the present moment. These constrained temporal policies may reflect empirical reports of the radically altered sense of time in deep meditation (Linares Gutiérrez et al. 2022).

With sufficiently reduced precision on visual beliefs, we increasingly enter into levels associated with formless experience, that is, experience not attributable to the five external senses or the body. Although object boundaries have already begun to be compromised by this point, the effect becomes more pronounced as we proceed into increasingly ‘objectless’ states. Where in the cortical hierarchy such processing occurs is unclear, yet the process appears to be beyond the visual system. This increasing (but not yet exclusively) objectless experience corresponds to territory in the ‘insight knowledge of equanimity’ and the fourth jhāna, since the structuring of objects has already considerably broken down in preceding stages, yet some lower-level visual object processing remains. Furthermore, reduced object processing is reflected in the reduced actionability and increased stillness or equanimity of this stage (Sayadaw 2016).

#### Substrates of objecthood

As the system continues the meditative deconstruction process, with a drastic reduction in higher-level content due to insufficient belief precision, moving experience and inference ever lower, we reach a level where beliefs appear to be concerned with spatial orientation itself. As discussed earlier, formless experience begins with a sense of unbounded spaciousness without form (i.e. no sensations attributable to the external world or the body) populating that space. This corresponds to the fifth jhāna and the knowledge of equanimity with the emphasis on spatial unboundedness (Sayadaw 2016).

We propose that the experience of unbounded space is due to insufficient precision over beliefs about very basic content. Information on spatial location provides necessary evidence for such content. The upshot is that attenuating precision over these low-level beliefs causes the system to move down the hierarchy to the underlying belief that spatial locational structure can best explain current sensory input. As space is perceived without contents within it, policies involving the spatial movement of attention (like a saccade to selectively sample a given area) would no longer be epistemically beneficial since there are no features within experience to be confirmed in spatial relation to others. Beliefs at this level would appear to correspond to a spatial prior or Kantian primal form of intuition, ‘space’ (Swanson 2016). Space, as a hidden cause or best explanation of the kinds of statistical regularities of input that creatures such as ourselves take in, may be an innate preference of the system—genetically imbued—or acquired early in life.^28^ Either way, Buddhists have long taken space to be a constructed phenomenon.^29^

Decreased precision over spatial-representation beliefs moves the system lower, as noted earlier, to beliefs which appear to represent an unbounded field of consciousness and presence. Such beliefs are experientially reflected with the prominence varying between the sixth jhāna, boundless consciousness, and the insight side, with the consciousness aspect of knowledge of equanimity.

Belief precision decreasing again moves further down to where lower-level sampling seemingly concerns an underlying basis upon which a sense of conscious presence is constituted. Here, beliefs concern the absence of any kind of presence in experience (corresponding to the seventh jhāna or nothingness aspect emphasized in knowledge of equanimity on the insight side).

An interpretation of this degree of deconstruction would be that this is hierarchically where beliefs underlying the capacity to conceptually grasp anything at all sit, as moving lower reveals phenomenal experience featuring complete conceptual ineffability. Similarly to above, where there is space without form, here there is absence without the presence of anything. Thus, at this level beliefs undergirding an experiential sense of presence appear to predominate. Decreasing belief precision at this level due to further release entrains information resampling at the next level, for the meditator, either the eighth jhāna (neither perception nor non-perception), or on the insight side, the neither perception nor non-perception aspect of knowledge of equanimity. As mentioned above, this level appears to concern beliefs which are reflected phenomenologically as ‘some experience’, yet in ineffable terms.^30^ Insufficient precision on these beliefs appears to result in an absence of phenomenal experience, discussed above as a cessation event over a very brief temporal window.

Thus, we propose cessation events are a consequence of insufficient precision on beliefs at even the lowest level of the hierarchy associated with phenomenal experience. In principle, epistemic policy selection, like before, would drive the process lower, yet there is no lower-level inference, at least which is associated with any experience at all. A full shutting down (albeit temporarily) of inference at any level associated with experience would mean no posterior beliefs about the best explanation for current sensory input that have phenomenal consequences.^31^ We suggest that a new theoretical-computational construct may be needed in the AIF literature to address this puzzle.

With regard to the neuroscientific understanding of cessation events, we note that Chowdhury et al. (2023) in an EEG study on an advanced meditator found cessation events to be associated with an alpha-power decrease in occipito-parietal areas of the brain. They note that the decrease in alpha power before cessation events are consistent with the hypothesis that such events are the consequence of attenuated hierarchical processing in the brain (p.15), given that alpha power is used as an index of top-down processing and high-level psychological functioning (Klimesch 2012, Walsh et al. 2020), while Sedley et al. (2016) found alpha-band oscillations relate quantitatively to prediction precision.

Thus, our account of the deconstruction process via the release in mental tension suggests that such tension underwrites experience itself. By repeatedly releasing mental tension, belief precision decreases at each level where release is enacted, driving epistemic foraging to progressively lower levels of the hierarchy. We have argued that the kinds of content encoded at these lower levels can account for the phenomenological changes witnessed during meditative deconstruction, including the absence of experience during a cessation event, when insufficient precision at the lowest level of the hierarchy associated with phenomenal experience results in the absence of any consciously reflected inference.

## Discussion

We developed a novel account of deep meditative deconstruction using Buddhist defabrication within the active inference framework. Constraining our examination to defabrication by an advanced meditator over a short temporal scale and assuming pre-existing skill, we identified the release of mental tension as a correlate of reduced clinging. This led to our proposal casting the reduction in mental tension as a level-specific reduction in belief precision.

**Figure 4:**
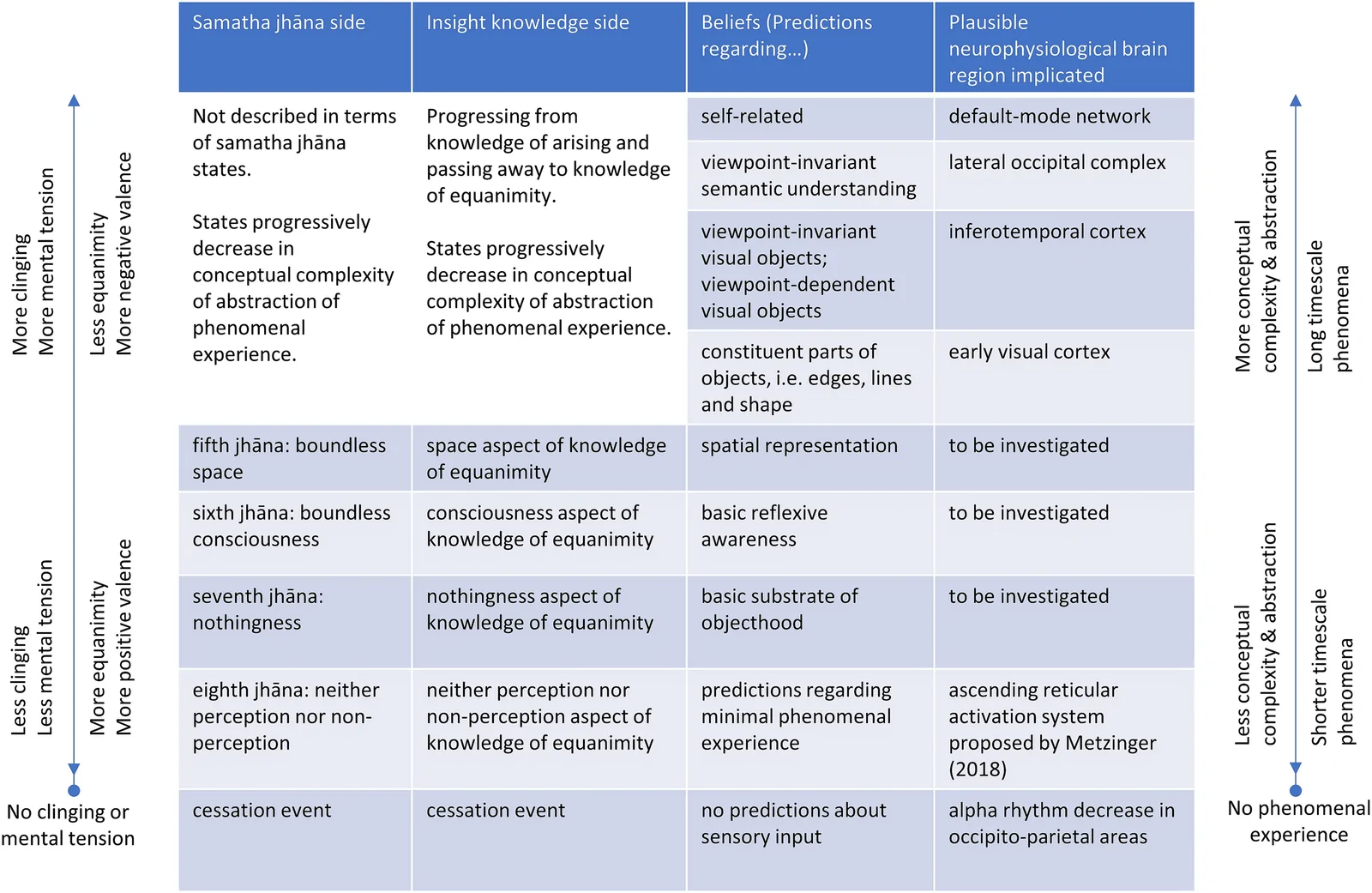
Full meditative deconstruction under AIF. Samatha jhāna (first column) and insight knowledge (second column) sides of defabrication as presented in illustrative example (Section Illustrative Example of Full Deconstruction), with associated beliefs (third column) attenuated via insufficient precision at that level, and corresponding reduced activity in plausibly implicated brain regions (fourth column). On the left: craving and mental tension decrease as deconstruction advances, with equanimity and positive affective valence increasing (left arrows). On the right: trends in conceptual belief complexity and duration of experiential phenomena (right arrows), ending in the absence of phenomenal experience with the cessation event (bottom row of table and rounded arrows). See Appendix for more on the presented correspondence between jhānas and insight knowledges. Entries marked ‘to be investigated’ reflect where region involved in such processing is an open empirical question.

We showed how the release of mental tension, due to expected free energy being scored in terms of both epistemic and pragmatic value, drives the system to lower-level inference in pursuit of epistemic value. When repeated ever-lower in the hierarchy, this results in a downward cascade of inference to progressively lower levels that feature decreased construction, abstraction and conceptual complexity in the meditator’s phenomenal experience (see Fig. 4). We then demonstrated that both increased equanimity and more positive affective valence correlate with this downward hierarchical traversal, offering a clearer AIF-based understanding of deep meditative experience.

Our illustrative example clarified the foundational role that low-level beliefs, and their specific hierarchical ordering, may play in serving as a substrate for higher-level beliefs about objecthood. This may serve to inform our understanding of the kind of core knowledge enabling higher-level comprehension and its structuring (Ullman and Tenenbaum 2020, Rorot 2021). We also examined cessation events in relation to hierarchical processing, finding them to be momentary yet complete disruptions in perceptual processing as it pertains to phenomenal experience, occurring when even the lowest-level beliefs lack sufficient precision to yield lived experience. We note that while the illustrative example we have provided here discusses defabrication predominantly with regard to the visual sense modality, defabrication occurs across all modalities including the auditory, somatic, interoceptive domains, thereby altering perception to feature increasingly deconstructed contents across them (for example, shorter time scale outer and inner speech and internal body representations).

One potential concern with our account is whether it is compatible with accounts where minimal phenomenal experience is associated with deep, organism-level generative processes regulating arousal and affect such as Solms and Friston (2018) and Ramstead et al. (2024). In those accounts, minimal experience is associated with foundational substrate layers of the generative model linked to the brainstem and sub-cortical systems. We believe this is broadly commensurate with our view, since as noted in Fig. 4, the minimal experience of eighth jhāna is at a level linked to the ascending reticular activating system in the brainstem (in our account after having traversed the levels associated with fine-grained sensory representations). This is also consistent with the idea, highlighted in the Solms and Friston account, that internal sensory input from interoception may have sub-cortical origins, while thalamic processing may play a role in the activation and deactivation of cortical regions discussed in our account. This would entail that subcortical processing could play a larger role in the lowest levels of conscious experience than presented here, where we have not made explicit distinctions between exteroceptive and low interoceptive levels. In the context of what these considerations may tell us about consciousness, an interesting question for further research is how to conceive the top versus the bottom in the cortical hierarchy, given the regulatory role of subcortical and brainstem activity, and the extensive subcortical–cortical loops that also characterize the hierarchy.

Another potential concern is that our account of meditative deconstruction could be considered similar to active inference accounts of autism, as both involve alterations in hierarchical processing and precision weighting resulting in increased low-level perceptual experience. While we acknowledge that both cases involve a certain phenomenological similarity—in the sense that perception features more hierarchically lower-level content—we emphasize that the proposed mechanisms differ. Autism, as interpreted by Lawson et al. (2014), involves permanently increased expected precision over sensory input, leading to increased prediction errors, decreasing the influence of high-level belief priors about hidden causes as an eventual downstream consequence. Therefore, in the case of autism, any potential decrease in high-level precision over beliefs is a consequence rather than an initial step of the condition, with the mechanistic route starting with permanently excessive precision over expected sensory input. In contrast, meditative deconstruction is a temporary, deliberate, and endogenous process. Initiated by reducing belief precision and pragmatic policy selection at the highest levels of the hierarchy currently activated, and then progressively attenuating belief precision and pragmatic policy selection at ever lower levels of inference. Importantly, there is no proposed direct alteration in expected precision over sensory input, as such increases would only be relative to the decreased influence of priors, as a downstream effect of higher-level attenuation. This means pragmatic policy selection has already been attenuated prior to the increase in sensory precision, preventing higher-level initiated pragmatic policy selection (behaviour) to fulfil preferences.

We recognize our account is merely a first step in integrating AIF, phenomenology and Buddhist understandings. One limitation is the phenomenological evidence supporting an advanced meditator’s experience, so future research would benefit from a more rigorous approach by employing micro-phenomenological interviews (Bitbol and Petitmengin 2017) to detail the deconstructive perceptual changes in more depth. Another limitation is the loose mapping between the earlier samatha jhānas, insight knowledges and the levels of beliefs attenuated in our example. While we have outlined this relationship in broad strokes, a more precise mapping requires a substantially deeper treatment, due to its complexity, than we are able to offer here, leaving this for future work. Likewise, since we take only initial steps toward an active inference account of meditative deconstruction, some areas remain underspecified, making this another limitation. For example, we failed to provide explanations of certain insight knowledges and formless jhānas in active inference framing, as they involve core knowledge structuring still under investigation. Nonetheless, we hope our approach inspires further exploration. Future research could develop a computational model of meditative deconstruction under AIF, formalizing and simulating its dynamics. Another avenue is exploring how deconstruction affects selfhood and identification, a complex but crucial area. Lastly, further work is needed to mechanistically explain how meditators engaging in Buddhist practices cultivate the capacity to deconstruct experience—a capacity we have assumed but not examined in depth. We hope this work lays the groundwork for these inquiries.

## Supplementary Material

Manuscript_Appendix_niag053

## Data Availability

Not applicable—no new data were generated or analysed in support of this work.

## Footnotes

1 Buddhist translations used here are from Pali and will be denoted via italics.

2 In Buddhist literature, ‘grasping’ is used in the same technical sense as clinging. We note that the process of clinging causes identification with aspects of experience, the sense of phenomena being ‘me’ or ‘mine’ (Burbea 2014). Due to limited scope identification is not further discussed in this work.

3 We use the terms negative or positive valence relative to one’s current valence in phenomenal experience. That is, positive valence refers to increasing the pleasantness of experience from what it currently is or just was, and negative valence refers to increasing the unpleasantness of experience from what it currently is or just was. We also use ‘more positive’ as meaning in a basic sense ‘preferrable to’ rather than referring to any particular texture of experience. For example, if a neutral experience is preferable to a happy one, then the neutral experience has more positive valence.

4 As a consequence, we do not discuss the ample literature on the cultivation of the capacity to deconstruct via meditation, under such terms as decentring (Bernstein et al*.* 2015), cognitive defusion (Fletcher and Hayes 2005), dereification (Lutz et al*.* 2015), opacification (Metzinger 2003), phenomenological reduction (as interpreted by Varela 1996), and experiential reduction (Katyal 2022). Similarly, we do not discuss the relationship between defabrication and meditative development (Galante et al*.* 2023).

5 We use ‘meditative defabrication’ to refer to the Buddhist process specifically, and ‘meditative deconstruction’ to refer to the AIF interpretation. This reflects the predominant use of ‘fabrication’ in Buddhist literature, and the more common employment of ‘construction’ in cognitive science and the AIF literature.

6 For more detailed introductions to AIF, see Friston (2010), Smith et al*.* (2022) and Parr et al*.* (2022).

7 Free energy minimization can be considered the same as the minimization of prediction error or information-theoretic surprise, under certain conditions (Parr et al*.* 2022).

8 We use ‘belief’ in the AIF sense of sub-personal beliefs encoded in probability distributions about the causes of sensory input, and not in the sense of conscious beliefs which are available to the system as thoughts in phenomenal experience. AIF represents beliefs about hidden causes of sensory input as probability distributions, with the precision (inverse variance) representing the confidence that a belief is true. For example, a categorical distribution may represent the probability of perceiving a car as 0.8 and a house as 0.2.

9 We emphasize that within modern technical formulations of AIF, hierarchical depth is functionally distinct from temporal depth (see Parr et al*.* 2022 for more) despite the notions often being conflated in earlier work in the field.

10 For general statements and introductions to the predictive processing framework, see Friston (2010), Hohwy (2013), Clark (2016).

11
Carhart-Harris and Friston (2019) propose that meditative and psychedelic states share psychological and neurobiological similarities in attenuating higher-level conceptual processing. In neuroimaging studies, both increase brain entropy—a quantitative measure of uncertainty about the state of a brain as a system (Carhart-Harris et al*.* 2014, Carhart-Harris 2018, Vivot et al*.* 2020, Kumar et al*.* 2021, Czajko et al. 2024, Chowdhury et al*.* 2025). Increased entropy is thought to reflect a key phase of the insight process. Decreasing precision of beliefs encoded at high-levels, such as an unchanging self (higher entropy) enables content previously hidden from experience by high precision priors to become perceivable. This permits new perspectives, with relaxed high-level priors allowing disinhibited information to travel up the hierarchy and be experienced as insights (Carhart-Harris and Friston 2019; p. 332). Relaxing high-level priors has also been linked to Zen Shikantanza practice (Pagnoni and Guareschi 2021) and open monitoring has been proposed to render lower-levels perceptible (Laukkonen and Slagter 2021, p. 205).

12 By precision, we refer to the inverse variance of a probability distribution and review the role of precision in more depth in Section Overview of AIF.

13 See Marchi and Hohwy (2022) and Nave et al. (2022) for a discussion of the (middling) areas and levels of the hierarchical generative model associated with conscious experience.

14 We do however acknowledge that what is considered an ‘advanced meditator’ is highly debatable, and are open to the possibility that what is considered advanced in one context might be seen as elemental in another. What we have at hand are claims, so we simply must utilize the apparently most reliable phenomenological claims, while acknowledging said claims may be over assumptions.

15 There are many other types of cessations not discussed here. These include partial, full or final extinguishment or liberation (*phala, Nibbāna*, or *parinibbāna*; Bodhi 2016; Sayadaw 2016, pp. 445–446). Nirodha-samāpatti (the attainment of cessation; Bodhi 2005, SN 28.9) and animittasamādhi, a cessation of any concepts (signs) by which something is recognized (Ñāṇamoli 1995; MN 43, MN121). Temporary yet protracted-cessations of elements of experience, yet not all experience, via recognition of the emptiness (*śūnyatā*) of perceptual phenomena (Ñāṇamoli 1995; MN 122; Nāgārjuna and Kalupahana 1991, p. 56, 266). *Bhavaṅga*, a state where no active conscious processing occurs which is either described as profound and positive (Collins 1982, p. 241–247), or as an obstacle to meditative development due to weak mindfulness (*asaññi-bhava;*  Ṭhānissaro 2006, p. 65), and even ‘wrong samādhi’ and wrong release (Lee Dhammadharo 1995).

16 As a general rule, we refer to Buddhist terms by their English translation, however, for some terms such as *samatha* and *jhāna* where we use the Pali source term, either because the translation has misleading connotations in English, such as for *samatha* or because it is in relatively common usage, such as for jhāna.

17 By ‘non-state’ we mean a state where there is no phenomenal experience.

18 The samatha jhānas are accepted by most Buddhist schools, while the insight knowledges (sometimes referred to as the stages of insight) are predominately employed Burmese Buddhism, and are not widely accepted in other Buddhist traditions (see Anālayo 2022, pp. 172–177), yet are argued to form a tractable schematic framework and developmental sequence (see Grabovac 2014).

19 See Figure A in the Appendix for a depiction of equivalencies between jhānas and insight.

20 While terms such as dissolution, fear, misery, disgust, desire for deliverance, might be perceived as negatively valenced, they are understood in the context of perceiving the drawbacks of grasping at phenomena which produce suffering (*dukkha*), which as outlined in Section Phenomenological variation within defabrication, produces positive valence.

21 There may be a third modality by which clinging is released, advancing the mind to a cessation event, by directly attempting to release any mental tension perceived in the mind. This route, targeted in practices like Do Nothing, Shikantaza (‘just sitting’) and Choiceless Awareness (Taft 2017, Young 2011).

22 See Prest (2021) for a predictive processing account of how this may plausibly occur.

23 We use the term ‘mental action’ as an action which does not engage the motor system.

24 Thus, our account differs from work by Laukkonen and Slagter (2021), Deane et al*.* (2020), Carhart-Harris and Friston (2019) among others because these accounts do not focus on mental tension and its release as a key phenomenological correlate of the deconstructive process.

25 See Parr et al*.* (2022, Sec. 2.8) for the precise mathematical formulation of the relationship between expected free energy (EFE), information gain and pragmatic value, yet put simply *EFE = − [Information gain]—[Pragmatic value]* (p*.* 33). Regarding preferences, technically, desired outcomes are encoded as positive prior preferences over observations and undesired outcomes as negative prior preferences.

26 Our account of meditative deconstruction, by leading to a reduction in hierarchical depth and consequently limiting temporal depth, renders this aspect of our proposal broadly in line with the Laukkonen and Slagter (2021) proposal that deconstruction reduces temporal depth, yet sets out in precise detail how and why this is the case, emphasizing the role of active inference in the process.

27 For example, Maha Bua (2012, p. 73) describes the process where a meditator who has gained proficiency finds their mindfulness and wisdom automatically working in unison without prompting, allowing them to investigate phenomena in successive stages with minimal effort. He goes on to suggest that there is an even deeper level of automaticity at the endpoint of the practice—supreme-mindfulness and wisdom—where both automatically drill down into everything experienced.

28 We do however acknowledge the need for further investigation of reports of the loss of the sense of spatiality in deep meditative states.

29 For example, see Burbea (2014) where he writes ‘a sense of the vastness of awareness is still itself an object, a perception, which includes, woven into it, a perception of space. [. . .] [T]he spectrum of fading reveals that any such perception of space is still fabricated. This appearance of a vastness of awareness is then a fabrication’. (Burbea 2014, Chapter 25. Emptiness and Awareness) or for a more traditional source see (Bodhi 2012; AN 9.34).

30 This may be a candidate for what Metzinger (2020) has termed minimal phenomenal experience. That is to say, we hold that the ineffable territory of eighth jhāna and the neither perception nor non-perception aspect of the knowledge of equanimity, due to being adjacent to cessation events, makes these the appropriate most minimally constructed experience on offer while still being phenomenal experiences and not the absence of such experience. Metzinger (2019) has proposed the Ascending Reticular Activation System may be implicated in such experience.

31 Lower levels of hierarchical processing beyond conscious experience appear to persist, as during cessations similar to those discussed here, autonomic processing, such as breathing and heart rate activity persists (see Laukkonen et al*.* 2023, p. 14).

## Abbreviations

AN, *Aṅguttara Nikāya* of The Buddha in Bodhi (trans.) (2012)

DN, *Dīgha Nikāya* of The Buddha in Walshe (trans.) (1995)

Dhp, *Dhammapada* of The Buddha in Norman (trans.) (1997)

MN, *Majjhima Nikāya* of The Buddha in Ñāṇamoli & Bodhi (trans.) (1995)

P, Pāli

SN, *Saṃyutta Nikāya* of The Buddha in Bodhi (trans.) (2005)
