# Supplementary material for: Towards an active inference account of deep meditative deconstruction
Source: Neurosci Conscious. 2026 Aug 24;2026(1):niag053. doi: 10.1093/nc/niag053 (PMC13502408; doi:10.1093/nc/niag053)
Supplement: Supplementary file 1 [file manuscript_appendix_niag053.docx]

# Appendix

In our account we employ a correspondence between the samatha jhānas and insight knowledges based on equivalencies by Pandita & Aggacitta (2012) and Ingram (2018). The correspondence in relation to defabrication is set out in Figure A, which also draws on work by previous work on the insight knowledges by Sayadaw (2016) and uses naming conventions from Grabovac (2014).

##### **Fig. A Two defabrication routes to cessation events**

Defabrication phenomenologically operates on a spectrum from left to right. Either jhāna (left column) or insight (right column) can be more or less prevalent at any moment (degree of weighting on the spectrum), but typically move downward via progressively reducing clinging by releasing mental tension (left arrow). This progressive release reduces fabrication (right arrow), moving phenomenal experience downwards towards the cessation event (bottom row). *Note that some samatha jhānas correspond to multiple insight knowledges (e.g., the third jhāna to insight knowledges from dissolution to fear), and some of the insight knowledges correspond to multiple samatha jhānas (e.g., the fourth to eighth jhānas correspond to the knowledge of equanimity).*
